# Structural changes in NOTCH3 induced by CADASIL mutations: Role of cysteine and non-cysteine alterations

**DOI:** 10.1016/j.jbc.2023.104838

**Published:** 2023-05-19

**Authors:** Soo Jung Lee, Xiaojie Zhang, Emily Wu, Richard Sukpraphrute, Catherine Sukpraphrute, Andrew Ye, Michael M. Wang

**Affiliations:** 1 Departments of Neurology, University of Michigan, Ann Arbor, Michigan, USA; 2 Neurology Service, Department of Veterans Affairs, VA Ann Arbor Healthcare System, Ann Arbor, Michigan, USA; 3 Departments of Molecular and Integrative Physiology, University of Michigan, Ann Arbor, Michigan, USA

**Keywords:** CADASIL, NOTCH3, gel mobility shift, conformational change, cysteines, disulfide bonds

## Abstract

Cerebral autosomal dominant arteriopathy with subcortical infarcts and leukoencephalopathy (CADASIL) is a cerebral small vessel disease that results from mutations in *NOTCH3*. How mutations in *NOTCH3* ultimately result in disease is not clear, although there is a predilection for mutations to alter the number of cysteines of the gene product, supporting a model in which alterations of conserved disulfide bonds of NOTCH3 drives the disease process. We have found that recombinant proteins with CADASIL NOTCH3 EGF domains 1 to 3 fused to the C terminus of Fc are distinguished from wildtype proteins by slowed mobility in nonreducing gels. We use this gel mobility shift assay to define the effects of mutations in the first three EGF-like domains of NOTCH3 in 167 unique recombinant protein constructs. This assay permits a readout on NOTCH3 protein mobility that indicates that (1) any loss of cysteine mutation in the first three EGF motifs results in structural abnormalities; (2) for loss of cysteine mutants, the mutant amino acid residue plays a minimal role; (3) the majority of changes that result in a new cysteine are poorly tolerated; (4) at residue 75, only cysteine, proline, and glycine induce structural shifts; (5) specific second mutations in conserved cysteines suppress the impact of loss of cysteine CADASIL mutations. These studies support the importance of NOTCH3 cysteines and disulfide bonds in maintaining normal protein structure. Double mutant analysis suggests that suppression of protein abnormalities can be achieved through modification of cysteine reactivity, a potential therapeutic strategy.

Cerebral autosomal dominant arteriopathy with subcortical infarcts and leukoencephalopathy (CADASIL) results from mutations in *NOTCH3* and is the most common monogenic cause of vascular dementia, stroke, and small vessel disease (1, 2). Over 100 disease-causing mutations have been described, with a vast majority of them encoding cysteine alterations in the NOTCH3 gene product (3, 4). Both loss or gain of cysteine residues in the NOTCH3 have been strongly associated with CADASIL; more specifically, mutations occur in the EGF-like domain containing extracellular region of this protein, a region of the protein that, by analogy to related protein structures, is scaffolded by over 100 intramolecular disulfide bonds. The bonding pattern within each EGF repeat involves cysteine 1→3, 2→4, and 5→6 connections in crystal structures (5). Consequently, disruption of the normal secondary structure of NOTCH3 could be the sentinel event leading to CADASIL vascular pathology.

Evidence for structural abnormalities in mutant NOTCH3 has been provided by investigations of recombinant NOTCH3 protein that has been produced in mammalian cells and analyzed *in vitro* (6, 7, 8). The most consistent differences between wildtype and mutant NOTCH3 proteins include (1) increased oligomerization of the mutant protein (6, 7, 8, 9) and (2) increased sensitivity of the mutant protein to *trans*-reduction by NOTCH3 peptides (6). Because NOTCH3 oligomers are sensitive to reducing agents, these properties of mutant NOTCH3 are compatible with a role of cysteines and disulfide pairing in the genesis of structural abnormalities in the protein.

Several features of mutant NOTCH3 that have not yet been clarified include (a) the relative impact and the range of cysteine mutations that causes NOTCH3 abnormalities (are there cysteine residue changes that do not cause disease), (b) the role of the amino acid residues that replace cysteines in loss of cysteine mutations in CADASIL, and (c) the role of unpairing of cysteines or free cysteines in CADASIL pathogenesis. Many of these questions are not answerable simply because mutations have not been described in patients yet. As such, analysis of the effects of an expanded range of mutations, generated *in vitro*, could facilitate a more detailed understanding of NOTCH3 protein alterations in CADASIL.

To this end, we describe a quantifiable and reproducible assay to assess alteration in NOTCH3 structure caused by CADASIL mutations. Because the assay is based on transfection of cells and can be scaled to assess mutations that have not yet been described and double mutations, we have been able to assess the role of a multitude of amino acid positions in NOTCH3 structure alterations. The results provide new insights into the range of mutations that change NOTCH3 structure, identify the role of specific amino acid residues on conformational changes, and shed light on the role of cysteine number and pairing in pathogenic NOTCH3 transformation.

## Results

### Upshifted NOTCH3 protein results from CADASIL mutations

**Figure 1:**
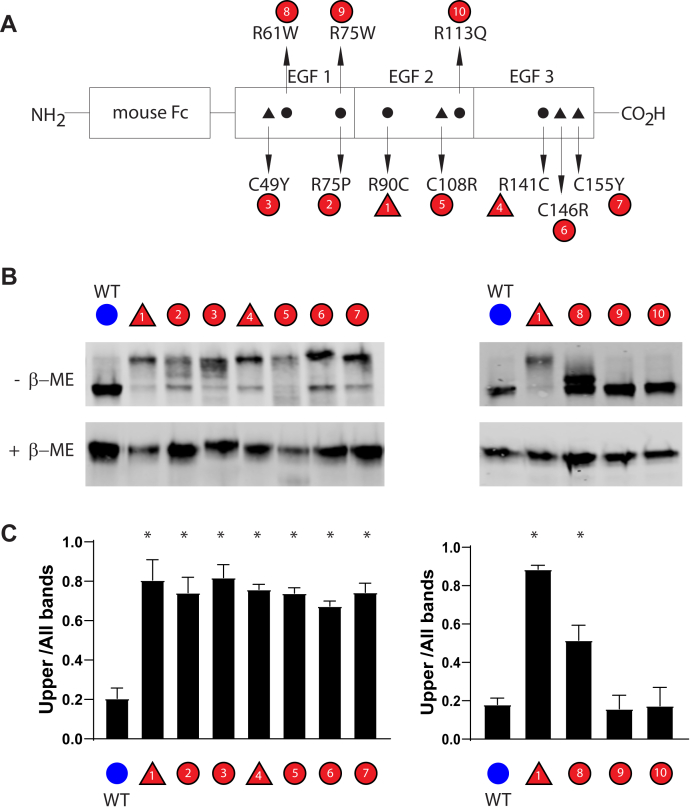
**Validation of gel shifting phenomenon with expanded set of pathogenic and nonpathogenic controls.** Protein from cells that were transiently transfected with Fc-NOTCH3(1–3) constructs were analyzed by Western blotting. *A*, schematic of constructs used in this set of experiments. Pathological variants that cause CADASIL are shown at the *bottom*. Variants listed in LOVD as benign are shown at the *top*. Cysteines are represented by *triangles*; non-cysteines are represented by *circles*; variants are in *red*. *B*, constructs were transfected into cultured cells and Protein A-agarose–bound proteins were separated without reducing agents on acrylamide gels without detergents. Comparison is made between wildtype recombinant protein and mutant recombinants that are labeled. The WT band is largely located in the faster running lower band; the location of most of the mutant protein is slower running upper bands. Variants that cause CADASIL were run on the left gel. Variants listed as benign in LOVD were run on the right gel, with WT and pathogenic proteins run alongside for comparison. On the *bottom* Western blots, the same proteins were separated after chemical reduction. All blots were probed with directly labeled anti-mouse antibodies. *C*, quantification of the ratio of upper band signal to total signal is shown and is the result of at least three independent experiments (∗*p* < 0.05 compared with wildtype control).

We generated fragments of NOTCH3 proteins to facilitate discovery of differences between WT and mutant polypeptides. After transient transfection of Fc-NOTCH3(1–3), recombinant construct composed of mouse Fc from IgG and the first three EGF-like repeats of human NOTCH3 (Fig. 1*A*), Protein A-agarose–enriched protein from conditioned media was isolated to assess the mobility of recombinant polypeptides. Western blot analysis using nonreducing gels showed that the wildtype NOTCH3 construct produced a protein of size expected for an Fc-linked dimer.

We then tested the effects of CADASIL mutations in NOTCH3 on the mobility of Fc-NOTCH3(1–3) recombinant protein (Fig. 1*A*, bottom, numbers 1–7). The mobility of seven CADASIL mutant proteins secreted into the conditioned media was assessed alongside wildtype Fc-NOTCH3(1–3) (Fig. 1*B*, left panel). CADASIL mutant NOTCH3 recombinant constructs directed the expression of Fc proteins that contained a distinct species that ran above the expected Fc-fusion dimer but faster than a double dimer. When the same protein preparations were reduced with beta-mercaptoethanol before gel electrophoresis, mutant proteins migrated with the same mobility as wildtype protein (Fig. 1*B*, lower panel). The proportion of Fc protein that was upshifted was significantly increased for all seven CADASIL mutants (Fig. 1*C*, left panel).

Three non-cysteine variants of NOTCH3 (Fig. 1*A*, top, numbers 8–10), annotated as nonpathogenic in the Leiden Open Variation Database (LOVD), were produced by transient transfection for comparison. Two of the nonpathogenic variants of NOTCH3 migrated in a pattern that was similar to wildtype NOTCH3, and none of the three mutants displayed the same magnitude of upshifting as the seven CADASIL mutants examined above (Fig. 1*B*, right). One of the mutants (R61W, number 8) displayed an intermediate degree of upshifting. On further review, R61W has been reported by some as a potential pathogenic mutant (10, 11). As before, when samples were reduced with beta-mercaptoethanol, the pattern of migration was the same for all variants (Fig. 1*B*, bottom). Quantification of these gel shifts is shown in Figure 1*C* (right panel).

The amount of gel shifting (normalized against total protein) did not show significant variation when different amounts of DNA was transfected. In Fig S1, multiple mutants produced similar ratios of shifted to nonshifted protein across a range of DNA used for all mutants tested. At the same time, the total amount of protein produced increased with increasing DNA. Thus, it appeared that gel shifting is a property of mutant NOTCH3 and not dependent on the quantity of protein produced.

These studies indicated that the reported pathogenicity of NOTCH3 mutations in EGF(1–3) correlates with the degree of upshift of the NOTCH3 expression product. Since the upshift due to mutations was reversed by beta-mercaptoethanol, altered gel mobility was likely a result of a disulfide bond–dependent conformation. We used the gel shifting assay in studies described below to explore the consequences of specific amino acid residues on NOTCH3 protein structure.

### Structural changes induced by loss of cysteine mutations in NOTCH3

**Figure 2:**
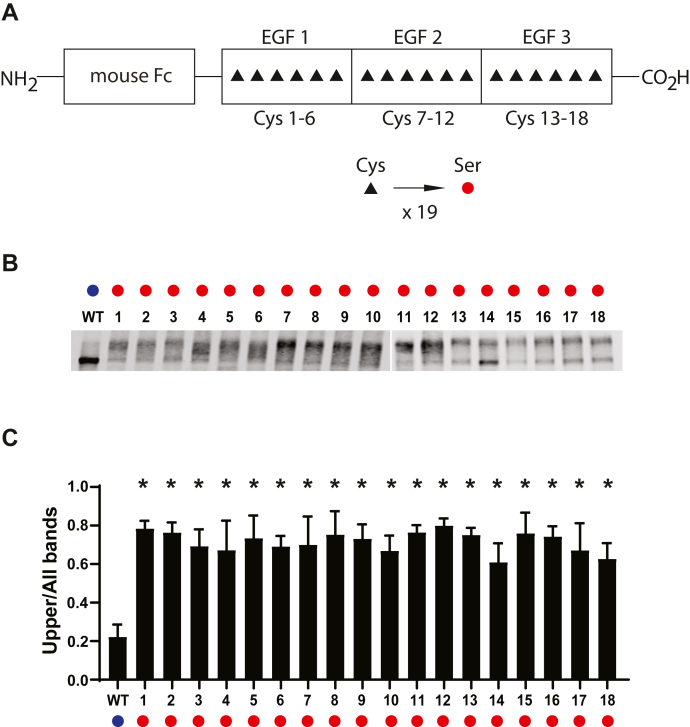
**Comparative effects of cysteine mutation location on NOTCH3 gel mobility.***A*, schematic showing sites of cysteines (*black triangles*) in the first three EGF repeats of NOTCH3 in the Fc-NOTCH3(1–3) construct. For this experiment, each of the 18 cysteines shown in NOTCH3 were mutated to a serine residue (*red circle*), resulting in 18 independent mutant clones, which were transfected into cultured cells, whose conditioned medium was analyzed for Fc protein mobility. *B*, Western blots of Protein A-agarose–enriched Fc-NOTCH3(1–3) proteins separated under nonreducing conditions. Wildtype protein was compared with 18 different mutants labeled 1 to 18. *C*, quantification of the ratio of upper band signal to total signal is shown and is the result of at least three independent experiments (∗*p* < 0.05 compared with wildtype control).

Although CADASIL mutations include a large range of mutations that mutate conserved cysteines, some loss of cysteine residues has not yet been reported as disease mutations. It is possible that mutations exist and have simply not been reported yet. Alternatively, it is possible that some cysteine residues are more likely than others to alter NOTCH3 structure. We queried the role of each of the cysteines in altering NOTCH3 structure by systematically altering each of the conserved cysteines in EGF-like domains 1 to 3. A total of 18 single cysteine-to-serine mutants were generated. As shown in Figure 2, we observed that all 18 cysteine-to-serine mutants demonstrated gel shift products. As such, it appears that loss of cysteine has an effect on NOTCH3 protein structure regardless of the location of the loss of cysteine within EGF(1–3).

### Effect of amino acid residue substitutions replacing conserved cysteines on NOTCH3 conformation

**Figure 3:**
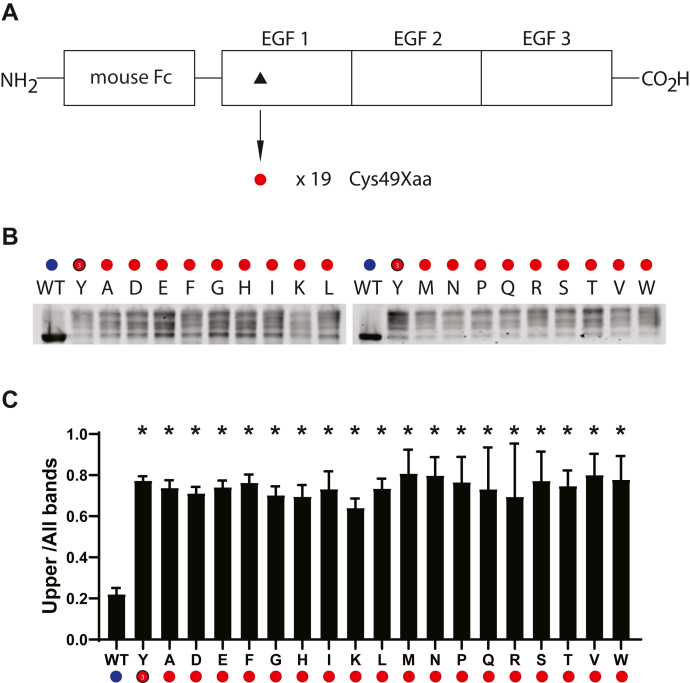
**Effect of amino acid substitutions at residue 49 of NOTCH3.***A*, schematic showing residue 49 of NOTCH3 within the first EGF repeat of the Fc-NOTCH3(1–3) construct. The C49Y mutant has been linked to CADASIL (Fig. 1). The WT residue is a cysteine residue (*black triangle*), which was mutated to all 19 non-cysteine amino acids (*red circles*), resulting in 19 independent mutant clones, which were transfected into cultured cells, whose conditioned medium was analyzed for Fc protein mobility. *B*, Western blots of Protein A-agarose–enriched Fc-NOTCH3(1–3) proteins separated under nonreducing conditions. Wildtype protein was compared with 19 different mutants labeled with the amino acid shown. Y represents the C49Y mutant. *C*, quantification of the ratio of upper band signal to total signal is shown and is the result of at least three independent experiments (∗*p* < 0.05 compared with wildtype control).

**Figure 4:**
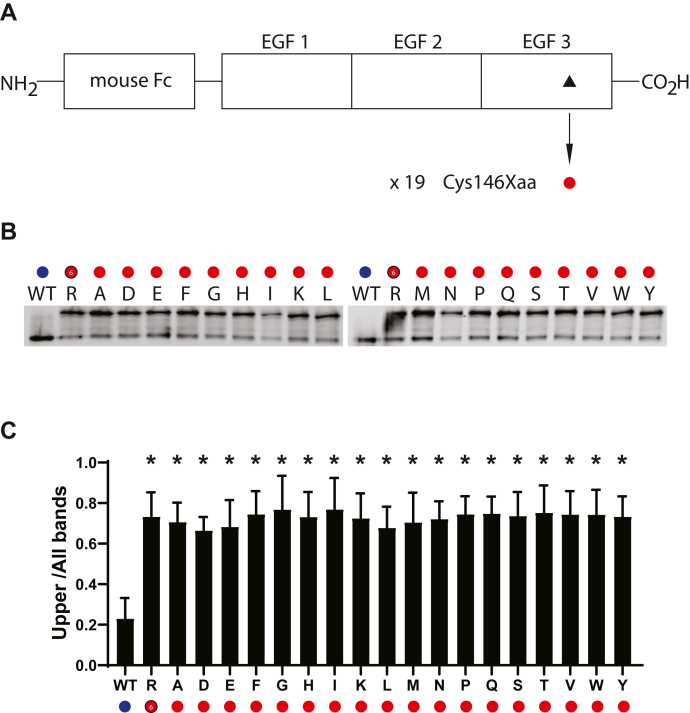
**Effect of amino acid substitutions at residue 146 of NOTCH3.***A*, schematic showing residue 146 of NOTCH3 within the third EGF repeat of the Fc-NOTCH3(1–3) construct. The C146R mutant has been linked to CADASIL (Fig. 1). The WT residue is a cysteine residue (*black triangle*), which was mutated to all 19 non-cysteine amino acids (*red circles*), resulting in 19 independent mutant clones, which were transfected into cultured cells, whose conditioned medium was analyzed for Fc protein mobility. *B*, Western blots of Protein A-agarose–enriched Fc-NOTCH3(1–3) proteins separated under nonreducing conditions. Wildtype protein was compared with 19 different mutants labeled with the amino acid shown. R represents the C146R mutant. *C*, quantification of the ratio of upper band signal to total signal is shown and is the result of at least three independent experiments (∗*p* < 0.05 compared with wildtype control).

Specific loss of cysteine mutations appears to frequently cause CADASIL. This may be due to restricted changes induced by single nucleotide changes. However, it is not currently known if there are specific amino acid residues that can replace cysteine without inducing structural changes. To compare the effect of all potential mutant amino acid residues, we generated a series of point mutation at positions 49 and 146, which are the positions of the CADASIL mutations C49Y and C146R. Mutant constructs were transfected and gene products were analyzed on nondenaturing gels to measure effects on the quantity of abnormally migrating protein. At position 49, all amino acid substitutions generated structural transformation of NOTCH3, with no appreciable differences between residues (Fig. 3). Similarly, at position 146, mutation of cysteine to any other amino acid resulted in upshifting of the protein product (Fig. 4). It appears that, for both positions 49 and 146, any mutation alters protein mobility, irrespective of the amino acid that replaces cysteine.

### Effect of different amino acid residues at position 90

**Figure 5:**
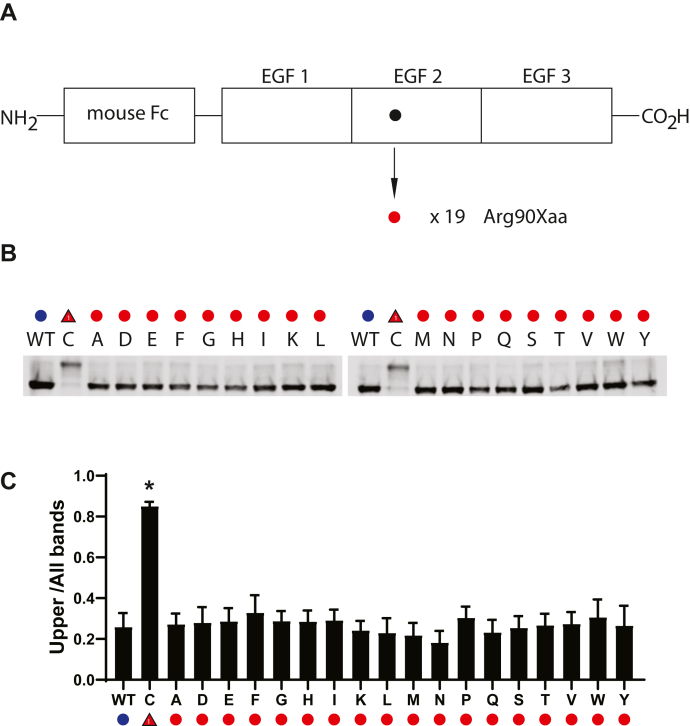
**Effect of amino acid substitutions at residue 90 of NOTCH3.***A*, schematic showing residue 90 of NOTCH3 within the second EGF repeat of the Fc-NOTCH3(1–3) construct. The R90C mutant has been linked to CADASIL (Fig. 1). The WT residue is an arginine residue (*black circle*), which was mutated to all 19 nonnative amino acids (*red*), resulting in 19 independent mutant clones, which were transfected into cultured cells, whose conditioned medium was analyzed for Fc protein mobility. *B*, Western blots of Protein A-agarose–enriched Fc-NOTCH3(1–3) proteins separated under nonreducing conditions. Wildtype protein was compared with 19 different mutants labeled with the amino acid shown. C represents the R90C mutant. *C*, quantification of the ratio of upper band signal to total signal is shown and is the result of at least three independent experiments (∗*p* < 0.05 compared with wildtype control).

The R90C mutant is a canonical NOTCH3 mutation that causes CADASIL. Mutations to other amino acid residues besides cysteine have not been described in control or CADASIL populations. To determine if structural changes were strictly associated with conversion to cysteine, we mutated residue 90 to all additional amino acids. Upshifting was highly specific for the gain of cysteine mutation, with none other mutant residue at position 90 capable of inducing upshifting of protein (Fig. 5).

### Effect of different amino acid residues at position 75 of NOTCH3

**Figure 6:**
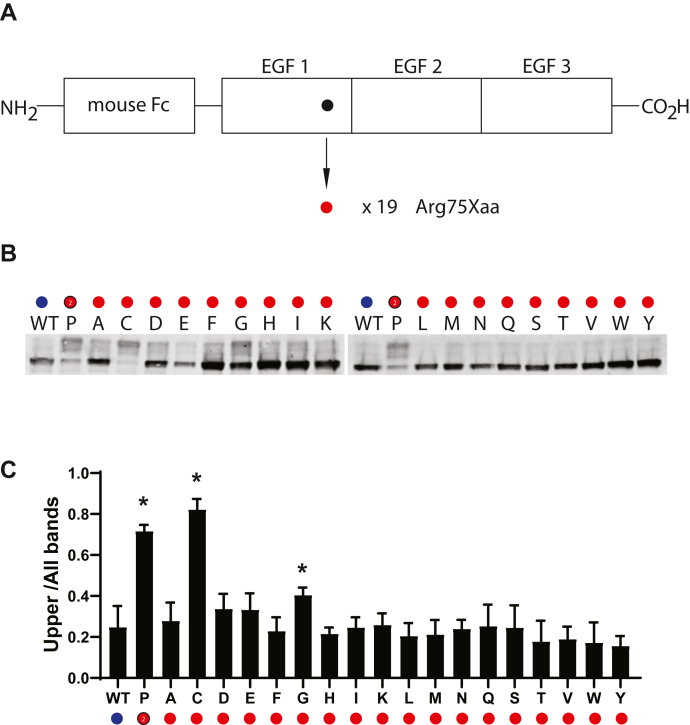
**Effect of amino acid substitutions at residue 75 of NOTCH3.***A*, schematic showing residue 75 of NOTCH3 within the first EGF repeat of the Fc-NOTCH3(1–3) construct. The R75P mutant has been linked to CADASIL (Fig. 1). The WT residue is an arginine residue (*black circle*), which was mutated to all 19 nonnative amino acids (*red*), resulting in 19 independent mutant clones, which were transfected into cultured cells, whose conditioned medium was analyzed for Fc protein mobility. *B*, Western blots of Protein A-agarose–enriched Fc-NOTCH3(1–3) proteins separated under nonreducing conditions. Wildtype protein was compared with 19 different mutants labeled with the amino acid shown. P represents the R75P mutant. *C*, quantification of the ratio of upper band signal to total signal is shown and is the result of at least three independent experiments (∗*p* < 0.05 compared with wildtype control).

Although canonical CADASIL mutations are cysteine-altering changes, uncommon non-cysteine residues have been described as causes of disease. One such change, R75P is a major cause of CADASIL in Korea and is hypothesized to result in protein conformation changes because of the helix-breaking nature of the substituted proline residue. Alternatively, the residue could be a key position that maintains structure whose alteration to any residue would alter structure. To differentiate between these possibilities, we mutated the R75 residue to all potential amino acids and applied gel shift analysis to the resulting recombinant proteins. Protein from transient expression was analyzed on nonreducing gels for gel shifts. Figure 6 shows that two amino acid substitutions besides R75P, R75C and R75G, were capable of causing gel shifting of protein, while all other residues resulted in a pattern of migration that was indistinguishable from the wildtype.

### Structural effects of cysteine substitution across EGF3 of NOTCH3

**Figure 7:**
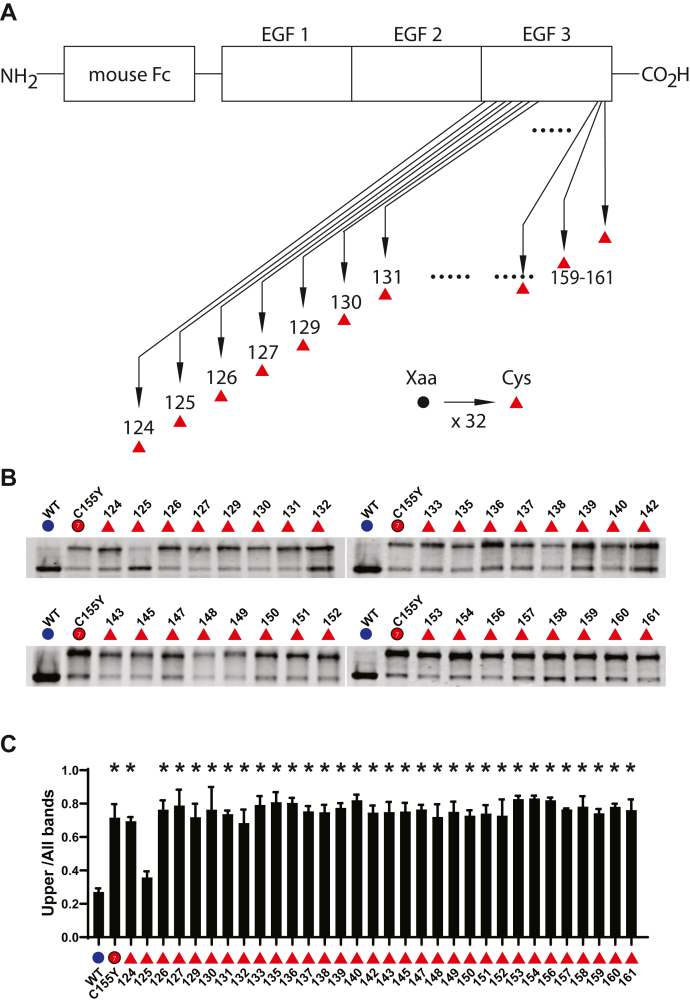
**Comprehensive analysis of cysteine substitutions in EGF repeat 3 of NOTCH3.***A*, schematic of scanning cysteine mutations introduced into the third EGF repeat of the Fc-NOTCH3(1–3) construct. Each non-cysteine residue was mutated independently to cysteine (*red triangles*), resulting in 32 independent mutant clones, which were transfected into cultured cells, whose conditioned medium was analyzed for Fc protein mobility. *B*, Western blots of Protein A-agarose–enriched Fc-NOTCH3(1–3) proteins separated under nonreducing conditions. Wildtype protein was compared with 32 different mutants labeled with the amino acid position shown. The C155Y mutant (Fig. 1; *red circle*) was also loaded for comparison. *C*, quantification of the ratio of upper band signal to total signal is shown and is the result of at least three independent experiments (∗*p* < 0.05 compared with wildtype control).

Substitution of cysteine residues into the N-terminal EGF-like domains of NOTCH3 are well-established causes of CADASIL. For example, in the third domain, pathogenic mutations occur when point mutations result in cysteines in residues S118C, G131C, R133C, R141C, F142C, G149C, Y150C, and R153C. Other gain-of-cysteine mutations have not been characterized or are considered variants of uncertain significance. To determine the range of cysteine substitutions within the third EGF-like repeat capable of causing structural changes in protein, we systematically mutated each non-cysteine residue to cysteine and assessed for gel shifting of recombinant protein (Fig. 7). We found that every mutation previously linked to disease (see above) demonstrated gel shifting. In addition, substitution of cysteine at all positions except residue 125 resulted in structural changes of the resulting gene product. Overall, with only a single exception, cysteine substitutions significantly affect NOTCH3 protein mobility.

### The effect of second cysteine mutations on NOTCH3 mobility

Because of the preponderance of CADASIL mutations that are cysteine altering, it has been proposed that pathology is triggered by changes in protein structure caused by an odd number of cysteine residues. An odd number of cysteines could induce changes that result from aberrant disulfide bonding within an EGF repeat, which normally contains three disulfide bonds. We used the gel shift assay to test the effect of second cysteine mutations, which results in an even number of cysteines. Some, but not all, second cysteine mutations affect residues that participate in disulfide bonds with a given CADASIL mutated cysteine residue; if two mutations in normally paired cysteines resulted in suppression of gel shifting, it would imply that a free, unpaired thiol is important for NOTCH3 conformational changes.

**Figure 8:**
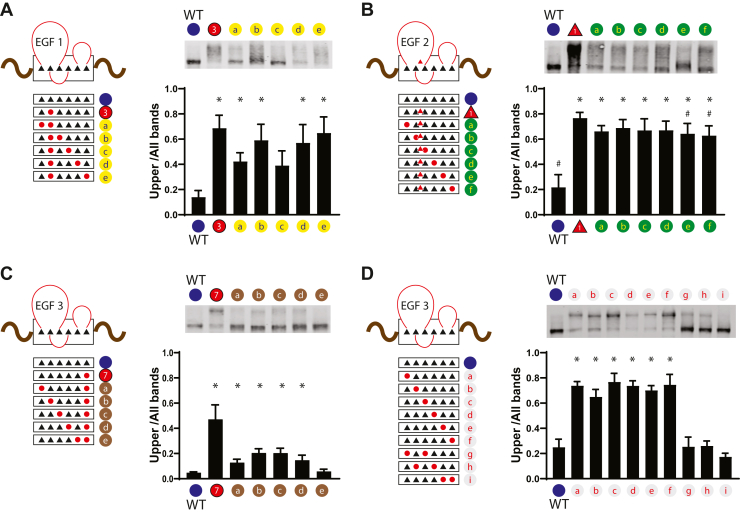
**Identification of suppressor mutations that reverse gel mobility abnormalities of NOTCH3 CADASIL mutants.** Three CADASIL mutant proteins in the Fc-NOTCH3(1–3) backbone were subjected to a second round of mutagenesis; each resulting double mutant harbors the original CADASIL mutation and an additional mutation that converts cysteine to serine. *A*, schematic of double mutants of C49Y, a CADASIL mutation in the first EGF repeat of NOTCH3, is shown in the *left panel*. The mutant cysteine normally bonds with the fourth cysteine in this repeat (see loops). Mutations are depicted in *red*. The *right panel* shows gel shift analysis and quantification. *B*, schematic of double mutants of R90C, a CADASIL mutation in the second EGF repeat of NOTCH3, is shown in the *left panel*. Mutations are depicted in *red*. The *right panel* shows gel shift analysis and quantification. *C*, schematic of double mutants of C155Y, a CADASIL mutation in the third EGF repeat of NOTCH3, is shown in the *left panel*. The mutant cysteine normally bonds with the fifth cysteine in this repeat (see loops). Mutations are depicted in *red*. The *right panel* shows gel shift analysis and quantification. *D*, schematic of double mutants in the third EGF repeat is shown in the *left panel*. The cysteine normal bond in a 1→3, 2→4, 5→6 configuration (see loops). Mutations are depicted in *red*. The *right panel* shows gel shift analysis and quantification. Results are from at least three independent experiments. Statistical analysis for differences between WT and mutants is shown with ∗*p* < 0.05; in (*C*) we also show statistical differences between the CADASIL mutant R90C and other groups #*p* < 0.05.

We first mutated individual cysteines in the presence of C49Y, which is a loss of cysteine CADASIL mutation (Fig. 8*A*). We changed each remaining cysteine in EGF1 individually in the presence of C49Y, a mutation of the second cysteine of the repeat. Protein encoded by these constructs were analyzed by gel shifting. We observed that mutation of the fourth cysteine, which is normally paired with C49, normalized the migration of the double mutant. Other double mutants, which harbor an even number of cysteines, exhibited abnormal shifting that was similar to the single mutant.

Second, we mutated individual cysteines in the R90C backbone, which is a gain of cysteine CADASIL mutation (Fig. 8*B*). On top of R90C, we mutated each of the six cysteines of EGF2 to serine. Protein from these six constructs were analyzed by gel shifting, and each demonstrated abnormal gel shifting similar to the parent mutant. There was a significant attenuation of gel shifting between the parent mutant and double mutants in which the fifth or the sixth cysteine of EGF2 was altered. This suggested that two of the cysteine thiols in the R90C mutant are important in conformational changes.

Third, we introduced cysteine-to-serine mutations in EGF3 in the presence of the C155Y mutation (Fig. 8*C*). As before, the single mutant C155Y protein exhibited gel shifting compared with the wildtype. The C155 residue is the sixth cysteine of the EGF repeat, which is normally disulfide bonded to the fifth cysteine of the repeat. When double mutants were tested in which the remaining five cysteines were mutated to serine, only mutation of the fifth cysteine mutant significantly decreased gel shifting.

In each of these examples, it is noteworthy that majority of constructs encoding even numbers of cysteines displayed conformational abnormalities. In addition, normalization of mobility shifting occurred preferentially when normally paired cysteines were mutated in tandem. To test the effects of additional altered pairs of thiols, we examined double mutants of EGF3 (Fig. 8*D*) by introducing serines for cysteines 1 and 3, and cysteines 2 and 4 (normally disulfide bonded cysteine pairs). These proteins exhibited normal gel migration patterns, while individual serine mutations exhibited aberrant shifting. Thus, suppression of gel mobility shifting by mutation of pairs that are normally disulfide bonded occurs with any of the three pairs of residues that participate in disulfide bonding. In Fig. S2, we found that addition of a nonspecific permeable cysteine alkylator iodoacetamide to cells producing the R90C mutant did not attenuate gel shifting of protein, which was consistent with cysteine suppressor specificity.

### Additional analysis of double mutations

To determine the specificity of the strongest suppressing cysteine mutations identified in Figure 8, we performed addition double mutation experiments. We compared the effects of the three suppressors from EGF1 (C65S), EGF2 (C117S), and EGF3 (C146S) on CADASIL mutations C49Y, R90C, and C155Y (located in EGF1, EGF2, and EGF3, respectively).

**Figure 9:**
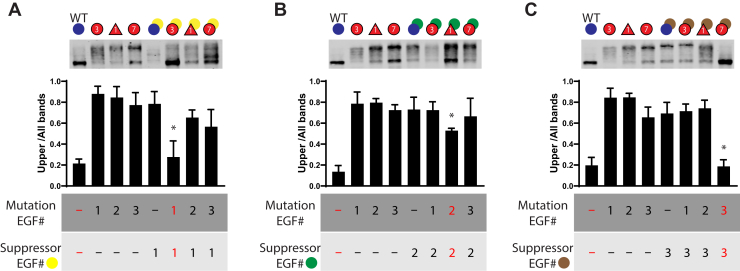
**Specificity of suppressor mutations for attenuating diverse CADASIL NOTCH3 mutations.** Suppressor mutants in EGF1 (C65S), EGF2 (C117S), and EGF3 (C146S) from Figure 8 were assessed for ability to normalize gel mobility shifts of CADASIL NOTCH3 mutations C49Y, R90C, and C155Y (located within EGF repeats 1, 2, and 3; see labels under each panel). We assessed if suppressors preferred to act on mutations within the same EGF repeat or, alternatively, were capable of acting across EGF repeats. *A*, effects of suppressor mutation C65S from EGF repeat 1. *Bottom labels* indicate the EGF repeat location of the suppressor and the CADASIL mutation. WT, single, and double mutation gene products underwent gel shift analysis (*top panel*) and quantification (*middle panel*). *B*, the same analysis as in (*A*), except that suppressor mutation C117S from EGF repeat 2 was tested in combination with three CADASIL mutations. *C*, the same analysis as in (*A*) but focusing on suppressor mutation C146S. Results are from at least three independent experiments. Comparisons were made between gel shifting of each single mutant (CADASIL NOTCH3 mutations) and the same mutant combined with a suppressor (three comparisons per panel); pair wise comparisons with ∗*p* < 0.05.

For suppressor C65S (Fig. 9*A*) located in EGF1, we found that mobility shifting persisted when this suppressor was combined with the R90C and C155Y mutations; these double mutants exhibited shifting comparable with that of the single mutants. As before, we observed that C65S suppressed the effects of the C49Y mutation from EGF1.

For the suppressor C117S (Fig. 9*B*), which decreased the abnormal mobility of R90C, combination with C49Y and C155Y failed to normalize mobility shifts. For the suppressor C146S (Fig. 9*C*) located in EGF3, the complete reversal of mobility shifting that was observed with EGF3 mutation C155Y was not observed for mutations C49Y and R90C, which are located outside of EGF3.

Overall, we did not observe suppression of protein shifting when cysteine mutations from different EGF repeat domains were combined. As such, the experiments of Figure 9 indicated that the suppressive effects of second cysteine alterations were specific for CADASIL mutations in the same EGF domain.

## Discussion

Extensive genetic analysis of *NOTCH3* in inherited cerebral small vessel disease has identified hundreds of pathogenic mutations as well as variants of uncertain significance (10, 12, 13, 14). Disease-causing mutations are almost all cysteine altering, with several exceptions such as R75P (8, 10). The protein analysis approach we describe herewith provides an opportunity to investigate the consequences of these mutations and to determine characteristics of mutations that have not been described in the population. In total, an unprecedented number of NOTCH3 variants (167) were analyzed. In line with all studies of CADASIL genetics, our investigation shows a highly important role of cysteine residues. (1) Without exception, mutations that resulted in the loss of a single cysteine produced aberrant NOTCH3 protein mobility. (2) The residue substituting for a conserved cysteine (at positions 49 and 155) does not appear to alter the degree of NOTCH3 protein mobility shift. (3) The mobility of NOTCH3 (EGF1-3) is quite tolerant to non-cysteine substitutions, with the exceptions of R61W, R75P, and R75G. (4) With only a single exception (S125C), mutations that result in gain of a single cysteine produced aberrant NOTCH3 protein mobility. (5) Replacement of pairs of cysteines that are normally disulfide bonded eliminates NOTCH3 protein mobility shifting.

The concordance between pathogenicity of mutations and abnormal gel mobility shifting is high. We compared pathogenicity annotated in LOVD for available mutants with gel shifting and found very high concordance between pathogenicity and mobility shifting. Conversely, lack of shifting of variants was strongly concordant with benign changes annotated in LOVD. Of note, we found that the mutation R61W, listed as benign in LOVD, has an intermediate level of gel shifting, although this suggested an inconsistency between gel shifting and pathogenicity; in fact, several reports have now implicated this mutation in CADASIL (10, 11).

The tight correlation between pathogenicity and the gel shift response of mutations provides good confidence that gel shifting novel mutants that have not yet been clinically described are likely to be associated with disease. Moreover, the assay offers an opportunity to investigate the impact of substitutions that have not yet been described in the population, yielding both predictive value and biochemical insight into the nature of CADASIL-causing mutations.

Although cysteine is the most significant residue responsible for aberrant gel shifting behavior of NOTCH3, we identify several context-dependent pathogenic residues. R61W, discussed above, induces alterations in protein mobility shifting. Moreover, the non-cysteine CADASIL mutant R75P migrates with an abnormal mobility like the canonical CADASIL cysteine mutants. No other amino acid besides cysteine (R75C) and glycine (R75G) at position 75 is capable of inducing protein abnormalities. The ability for proline to cause protein shifts appears to be selective for residue 75, since other mutants in which proline is introduced (R90P [Fig. 4]) did not exhibit abnormal protein migration. SNP databases list multiple proline variants of NOTCH3; future studies using gel shifting assays could be executed to investigate the biochemical effects of proline variants across NOTCH3.

One limitation of this study is that it is still not clear how NOTCH3 gel shifting occurs, but there are several clues presented regarding this. Gel shifting appears to strongly align with cysteine mutations. Another clue is that differences between wildtype and mutant proteins are eliminated by chemical reduction of protein. An idea that is consistent with these observations is that the NOTCH3 protein may be forming aberrant intramolecular disulfide bonds shortly after synthesis that distort the normal protein structure, leading to abnormal mobility. The intramolecular bonds could occur across the dimerized Fc protein, within the NOTCH3 chain, or even between Fc and the NOTCH3 portion of the protein. More detailed characterization will be needed to decipher the biochemical mechanism of NOTCH3 gel shifting. Of note, gel shifting was observed in three independent cell lines (Fig. S3). Another limitation of this design is that it does not easily permit study of full length NOTCH3 ectodomain due to the large size of the protein that makes it difficult to discriminate gel-shifted species; alternative separation techniques are required for future studies of larger NOTCH3 fragments.

Although the mechanism of the gel shift associated with pathogenic NOTCH3 variants is not certain, we believe the approach taken here can be extended to provide additional insight into mutant NOTCH3, which appears in over 1:500 individual genomic sequences according to two general population studies (15, 16). It is also unclear if C-terminal mutations are biochemically distinct from N-terminal mutations in NOTCH3, which is implied by the increased pathogenicity of mutations in the first six EGF repeats (17, 18); gel shifting of mutants outside of EGF 1 to 3 could be employed to study the impact of mutations in other regions of NOTCH3. In addition, the gel shift assay may be useful to investigate variants of uncertain significance in a wide range of disease states in which mutations are found in EGF-like domains of other genes, such as Marfan syndrome (19).

Finally, experiments with double mutations in cysteines have provided insights into the molecular requirements for gel mobility shifts. Second cysteine mutations attenuate protein alterations, specifically when the second cysteine resides in the same EGF as the CADASIL mutation. Furthermore, mutations in two nonpaired cysteines, rendering an even number of cysteines, has been shown in many examples to be incapable of blocking gel mobility shifting. These observations refine our understanding of CADASIL protein alterations in two ways. First, the assertion that an odd number of cysteines equates with protein pathology appears to be an oversimplification, since even numbered cysteine mutants exhibit gel shift abnormalities (Fig. 9). Second, the elimination of a disulfide bond within an EGF repeat is not sufficient to induce protein abnormalities (Fig. 9). This casts doubt on the model that decreasing disulfide scaffolding is the sole driver of NOTCH3 alterations.

Instead, a model of pathogenesis that aligns with our observations is that CADASIL is triggered by the presence of a free, unpaired thiol that results from impaired disulfide bonding. The unpaired thiol may be available to react with other molecules, including NOTCH3, which may drive protein oligomerization. The beneficial effects of deletion of specific thiols on protein mobility implies that, in a given mutant protein, specific residual thiols mediate NOTCH3 abnormalities. Furthermore, if this model is correct, it may be feasible to target unpaired thiol residues as a therapeutic strategy.

## Experimental procedures

### Generation of molecular constructs

The Fc-NOTCH3(1–3) construct is composed of mouse Fc fused to human EGF-like repeats 1 to 3 and has been described before (6). Point mutations were generated by PCR-directed mutagenesis using primers harboring specific mutations followed by ligation of mutated sequences into the parent vector (20). In cases in which a series of mutations was required, degenerate primer mixes were used. Single clones were sequenced for confirmation. Subsequently, DNA was prepared from colonies for transfection into cultured 293 cells.

### Gel mobility shift assay

Plasmid DNA encoding Fc-NOTCH3(1–3) and point mutants were transfected into 293 cells grown in six-well plates using lipid-based reagent (PolyJet; SignaGen) according to the manufacturer’s protocol (6). Conditioned medium (Opti-MEM; Gibco #31985-070) was collected after 24 h of transfection and incubated for up to 18 h with Protein A-agarose beads, which were washed and then eluted in sample buffer (50 mM Tris, 2% SDS, 0.1% bromophenol blue, 10% glycerol) without reducing agents, unless specified. Protein A–captured material was run on 4 to 20% Tris-glycine PAGE gels without added detergent (Novex; XPO4205BOX) in the running buffer contain 25 mM Tris, 190 mM glycine, and 0.1% SDS. The gels were transferred to nitrocellulose membranes using an iBlot 2 system. The membranes were then probed with infrared fluorophore-labeled secondary antibodies against mouse IgG (Li-Cor) and detected and quantified using a Li-Cor Odyssey infrared scanning system. The magnitude of gel shifting was defined as the amount of protein running above the wildtype Fc-NOTCH3(1–3) band compared with the total (above the WT band + the WT band).

### Statistics

Normality was determined by Shapiro–Wilt test. When appropriate, significant differences were determined using one-way ANOVA with Dunnett’s multiple comparisons test using GraphPad Prism v.7.0 c. A *p* value < 0.05 was considered statistically significant.

## Data availability

Experimental data and NOTCH3 cDNA plasmids generated for this study are available upon request.

## Supporting information

This article contains supporting information.

## Conflict of interest

The authors declare that they have no conflicts of interest with the contents of this article.
