# Supplementary material for: Structural changes in NOTCH3 induced by CADASIL mutations: Role of cysteine and non-cysteine alterations
Source: J Biol Chem. 2023 May 19;299(6):104838. doi: 10.1016/j.jbc.2023.104838 (PMC10318516; doi:10.1016/j.jbc.2023.104838)
Supplement: Supporting Figure S1 [file mmc1.pdf]

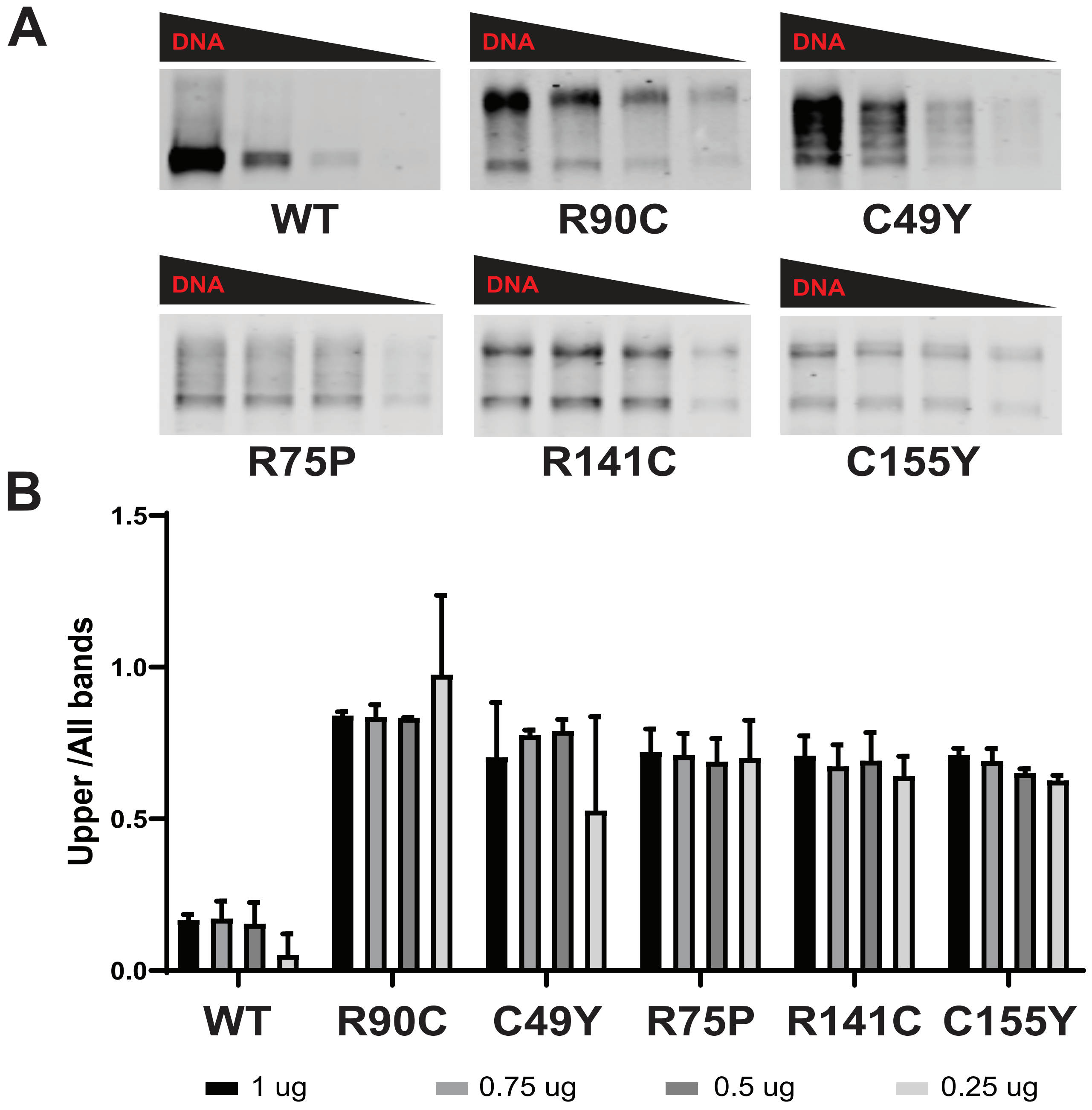

Supplemental Fig 1. Effect of protein production on gel shifting of NOTCH3 recombinant proteins. Fc-NOTCH3(1-3) (wildtype [WT] and mutants shown) constructs were transfected into 293 cells at the following doses: 1, 0.75, 0.5, and 0.25 ug. Empty vector was added such that the total DNA was 1 ug per well in a 6 well plate. Protein from conditioned media was concentrated using Protein A- agarose and analyzed on non-reducing gels by immunoblotting for Fc, as described in methods. (A) Representative immunoblot probed for Fc is shown. (B) Quantification of gel shifted Fc protein normalized to total Fc protein is shown below. The experiment was repeated two times. None of the lower dose ratios were statistically different from the full dose transfection (1 ug/well).
