# Supplementary material for: Structural changes in NOTCH3 induced by CADASIL mutations: Role of cysteine and non-cysteine alterations
Source: J Biol Chem. 2023 May 19;299(6):104838. doi: 10.1016/j.jbc.2023.104838 (PMC10318516; doi:10.1016/j.jbc.2023.104838)
Supplement: Supporting Figure S2 [file mmc2.pdf]

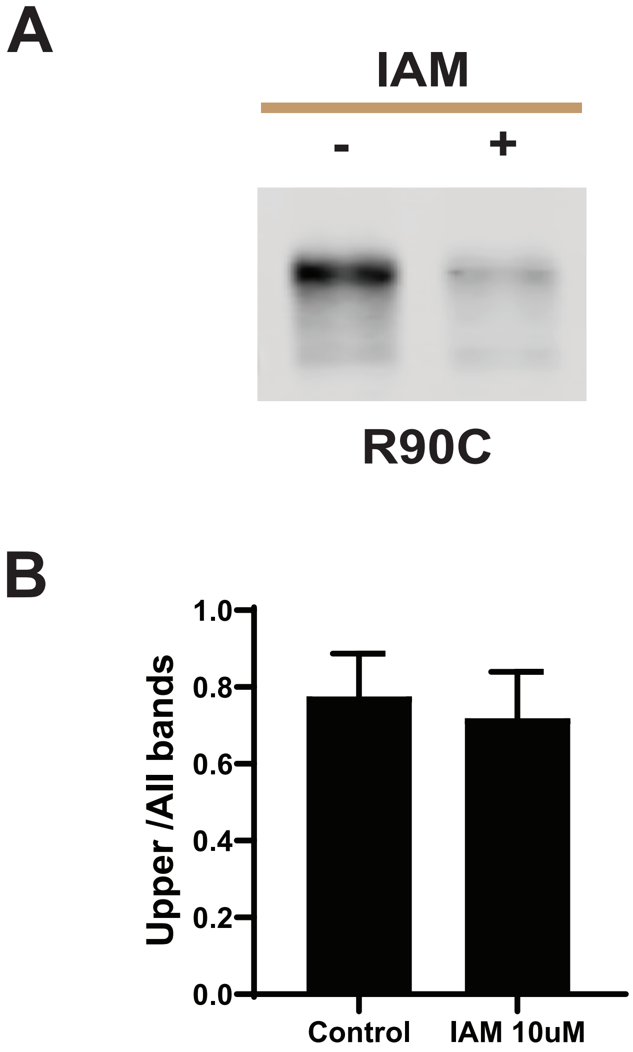

Supplemental Fig 2. Effect of iodoacetamide on NOTCH3 gel shifting. The Fc-NOTCH3(1-3) R90C construct was transfected into 293 cells and after an overnight incubation, the media was replaced with fresh OptiMEM or OptiMEM supplemented with 10 uM iodoacetamide for six hours. Protein was concentrated using Protein A- agarose and analyzed on non-reducing gels by immunoblotting for Fc, as described in methods. (A) Representative immunoblot probed for Fc is shown. (B) Quantification of gel shifted Fc protein normalized to total Fc protein is shown below. The experiment was repeated four times. There were no statistically significant differences between the groups. Higher doses of iodoacetamide yielded unacceptable toxicity after 2 hours and yielded too little protein for this assay.
