# Supplementary material for: Structural changes in NOTCH3 induced by CADASIL mutations: Role of cysteine and non-cysteine alterations
Source: J Biol Chem. 2023 May 19;299(6):104838. doi: 10.1016/j.jbc.2023.104838 (PMC10318516; doi:10.1016/j.jbc.2023.104838)
Supplement: Supporting Figure S3 [file mmc3.pdf]

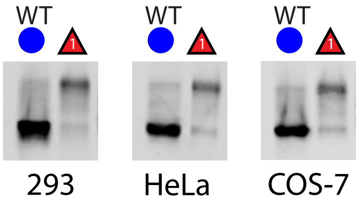

Supplemental Fig 3. Gel shift analysis of NOTCH3 recombinant protein expressed in multiple cell lines. Wildtype and CADASIL R90C mutant Fc-NOTCH3(1-3) protein was produced by transfection into 293, HeLa, and COS-7 cells. Conditioned media was analyzed on non-reducing gels, as described in Fig 1. The blue circles show wildtype (WT) protein and the red triangles indicate R90C mutant protein.
